# Abcès de l'avant bras: penser à l'ostéomyélite, même chez un adulte immunocompétent

**DOI:** 10.11604/pamj.2015.21.130.7090

**Published:** 2015-06-16

**Authors:** Ines Kechaou, Imène Boukhris

**Affiliations:** 1 Service de Médecine Interne B, Hôpital Charles Nicolle, Tunis, Tunisie

**Keywords:** Ostéomyélite, adulte, os long, immunocompétent, osteomyelitis, adult, long bones, immunocompetent

## Image en medicine

L'ostéomyélite est classiquement décrite chez l'enfant. Elle est la conséquence de la contamination de l'os, par voie hématogène, par un ou plusieurs microorganismes. Elle affecte habituellement la métaphyse des os longs des membres inférieurs. L'affection est rarement rapportée au-delà de 18 ans. Quand elle survient chez l'adulte, le plus souvent elle fait suite à une contamination directe post chirurgicale ou après une fracture ouverte, ou par atteinte par contiguïté. Dans d'autres cas, elle survient sur un terrain de patients immunodéprimés ou drépanocytaires. Nous rapportons une observation exceptionnelle d'une ostéomyélite survenant chez une adulte, immunocompétente, survenant sans circonstances favorisantes et atteignant un membre supérieur. Il s'agissait d'une patiente âgée de 23 ans, sans antécédents, qui était admise pour fièvre aigue chiffrée à 40°C avec douleur et tuméfaction cutanée d'aggravation progressive au niveau de la face antérieure de l'avant-bras droit. Le bilan biologique avait objectivé une hyperleucocytose à 23000 éléments (à prédominance de PNN). Elle avait un syndrome inflammatoire biologique avec une CRP à 150 mg/l, une VS à 100mm à H1, une fibrinémie à 5g/l et une anémie inflamamtoire à 10 g/dl d'hémoglobvine. L’échographie des parties molles, refaite à 3 reprises, n'avait pas montré de collection. Devant l'aggravation des signes inflammatoires locaux faisant évoquer un abcès sous cutané et la discordance entre la clinico-radiologique, une IRM était réalisée, montrant une image centromédullaire en hyposignal T1 avec rupture de la corticale au niveau du radius droit et issue d'une collection au niveau des parties molles. Le diagnostic d'ostéomyélite était retenu.

**Figure 1:**
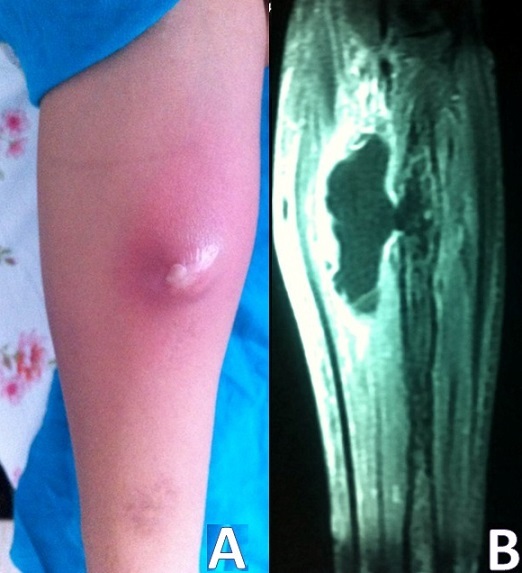
(A): lésion inflammatoire de l'avant-bras droit évoquant un abcès sous cutanée; (B): image centromédullaire en hyposignal T1 avec rupture de la corticale au niveau du radius droit et issue d'une collection au niveau des parties molles en rapport avec une ostéomyélite

